# The Effect of Selection Bias in Studies of Fads and Fashions

**DOI:** 10.1371/journal.pone.0123471

**Published:** 2015-04-17

**Authors:** Jerker Denrell, Balázs Kovács

**Affiliations:** 1 Warwick Business School, Coventry, United Kingdom; 2 Institute of Management, Universita della Svizzera italiana, Lugano, Switzerland; Universiteit Utrecht, NETHERLANDS

## Abstract

Most studies of fashion and fads focus on objects and practices that once were popular. We argue that limiting the sample to such trajectories generates a selection bias that obscures the underlying process and generates biased estimates. Through simulations and the analysis of a data set that has previously not been used to analyze the rise and fall of cultural practices, the New York Times text archive, we show that studying a whole range of cultural objects, both popular and less popular, is essential for understanding the drivers of popularity. In particular, we show that estimates of statistical models of the drivers of popularity will be biased if researchers use only trajectories of those practices that once were popular.

## Introduction

Cultural objects, phrases, names, and organizational practices rise and fall in popularity and researchers in sociology, psychology, and management have tried to understand the underlying dynamics [1–6]. External influences such as political and cultural events can sometimes explain why novel concepts and new organizational practices become popular [7]. But as many social scientists have noted, popularity also has its own dynamic and changes in popularity may be inherent in the process by which popularity is constructed [8–9, 3]. Popularity can beget popularity because people are more likely to discuss or get exposed to popular concepts [10]. The opposite can also be true with individuals who aspire to be fashion leaders actively avoiding restaurants and shops that may be considered to be excessively popular [11].

Despite the interest in the topic, there is little systematic research that tracks both the rise and the fall in popularity and also the full range of popularity, from the very popular to the unknown (for exceptions, see [5, 12–14]). Most research on the spread of ideas and practices covers only the process of diffusion and pays little attention to abandonment, either empirically or theoretically [4]. In addition, most empirical studies focus on practices and objects that are or once were popular [15–17]. Even research that is explicitly focused on fads and fashion often fails to cover the full range of popularity. While empirical studies on fads and fashions [18, 2, 19] cover the rises and falls in popularity, they suffer from a different selection bias. Typically, studies of fads focus on objects and practices that once were popular. For example, management researchers examine the rise and fall of quality circles and total quality management [2, 19] but there is little attention to the numerous objects and practices that never became very popular, possibly because it is more difficult to obtain data on objects and practices that were never popular, and in cases of the lowest popularity, researchers might not be aware of them at all.

We assert that understanding the dynamics of popularity requires an examination of more than the trajectories of a select set of practices that once were popular, such as quality circles. Limiting the sample to such trajectories generates a selection bias that obscures the underlying process and generates biased estimates. Specifically, we show that estimates of statistical models of the drivers of popularity will be biased if researchers use only trajectories of those practices that once were popular. To demonstrate this, we use simulations as well as a data set that previously has not been used in diffusion research.

Using simulations we show that coefficient estimates of the drivers of popularity will be biased if researchers only select trajectories of concepts and practices that once were popular. In particular, we show that in a selected sample the effect of increases in popularity is overestimated. Even if past increases in popularity do not impact on popularity in subsequent periods, past increases may appear to have a significant and positive effect on popularity in subsequent periods [5], thus providing a spurious ‘momentum’ effect. Moreover, the negative effect of competition will be underestimated in a select sample. We also show that both the effect of age and the effect of delays are underestimated in select samples.

Using a data set that enables us to track the popularity of a more complete range of novel concepts, we validate our simulation findings and show, for example, that the effect of increases in popularity effect is overestimated empirically. Following prior contributions [2, 19], we use media mentions to track the popularity of novel concepts and practices. Unlike prior contributions, however, we examine both the popular and less popular practices. Specifically, we analyzed the text archive of the New York Times from 1985–2006, and tracked the trajectories of the frequencies of individual words to measure their popularity (high frequency of mentions signals high popularity). A text corpus such as the New York Times Archive is suitable for our purposes because it contains a variety of concepts, cultural objects and practices. Because data exist over time on the frequency of all words, it is possible to examine whether estimates of diffusion and abandonment processes are biased if one only studies words that became popular but then declined in popularity. We restrict the set of words we track to those words that have not appeared before 1990, thereby ensuring that all the concepts are novel and avoiding the problems posed by left censoring [20]. By running regressions on the full data set, as well as on selective samples, we examine empirically the impact of selection bias on coefficient estimates. Like the simulations, the regression results show that the effect of increases in popularity is overestimated and the effect of competition underestimated in a select sample.

The rest of the paper is structured as follows. First, we introduce our data set, the New York Times archive—we start with the description of the data set because this data set provides a powerful demonstration of how trajectories of cultural concepts, objects, and practices follow a variety of patterns, and not just the patterns more frequently studied by scholars, such as a continuously increasing popularity or a fashion-like trajectory. As we demonstrate, most objects never become popular; thus models that describe the dynamics of popularity need to account for these processes as well. Second, we review the existing theories, models, and frameworks that set out to explain the dynamics of popularity, and discuss the extent to which tests of these theories will be biased if researchers selectively sample trajectories of objects that increased and then decreased in popularity. Third, we examine how selection bias would impact statistical estimates of the drivers of popularity. We analyze our proposed models using Monte Carlo simulation in order to examine the biases that might arise by focusing on popular or fashion-like phenomena. Then we return to the empirical setting and corroborate the simulation results by estimating the proposed model on the New York Times data. We conclude with a discussion of the findings and their implications for theory and research on the dynamics of popularity.

## Data

If most diffusion studies suffer from the shortcoming of limiting their focus to popular phenomena, what popularity trajectories do these studies miss? To answer this question, one needs to study a comprehensive set of diffusion trajectories. Admittedly, one of the main challenges in doing so is the difficulty of obtaining data on phenomena that did not become popular. In some settings, however, getting the full risk set is possible by analyzing natural imprints of social life, such as newspaper archives.

In this paper, we analyze such an archive: the text archive of the New York Times from 1985–2006. Specifically, we analyze the trajectories of the frequencies of individual words. A text corpus such as the New York Times Archive is suitable for our purposes because it contains a variety of trajectories. As data exist over time on the frequency of all words, it is possible to examine whether estimates of diffusion and abandonment processes are biased if one only studies words that became popular but then declined in popularity.

Media mentions and word counts have often been used to study diffusion and abandonment processes. For example, [2] analyzes the diffusion and abandonment of quality circles through tracing media mentions. Similarly, [19] trace the spread of Total Quality Management through the number of times it was mentioned in the ABI/Inform archive. The rationale behind using media mention counts to measure the diffusion and abandonment of practices is straightforward: usage and interest in a phenomena are mirrored in written text, so by following text mentions one can make inferences on the underlying popularity of the phenomena [21]. Admittedly, media mentions do not necessarily reflect usage: an established management practice that is no longer novel might not be mentioned very often in the business press but may still be frequently used. It would have been ideal to have data on how many individuals or firms that use a given management or cultural practice. It is very difficult to obtain such data for a wide range of practice, especially practices that did not become popular. As a result, we follow prior empirical studies of fads and rely on media mention as a proxy for usage and interest.

While the number of media mentions is often used to examine the dynamics of popularity, most studies limit their sample to one or a restricted number of trajectories, selected because they represent currently or formerly popular phenomena. Here we wish to avoid using such a selected sample. For this reason, we go to the other extreme and sample all trajectories rather than selected trajectories of media mentions that are known to rise and fall. That is, rather than identifying a subset of words or phrases (such as “Quality Circles” or “Total Quality Management”) and then track media mentions to these words or phrases over time, we examine word counts for all words. That is, we chose the unit of analysis to be a word, in this case a string of characters that appear between two spaces (or between any two so-called stopping character, such as,.! ?”). Following this procedure, we identified 1,177,482 unique words, the trajectories of which we followed from 1985 to 2006. We aggregate the word counts by years and these data allow us to compare estimates based on all words and estimates based only on words that went in and out of fashion.

A problem with the data we have is that it is left-censored: the trajectories of many words started well before 1985, but we do not have information about those word counts. To circumvent this problem, we have selected the words that have a zero count in the first five years of the archive, that is, words that were not mentioned before 1990. This approach gives us a risk-set: new words that had the potential to catch on but did not necessarily catch on (they just have to satisfy the sample’s selection criteria, i.e., the word has to be mentioned at least once between 1990 and 2006). Selecting words that have not appeared before 1990 results in a sample of 662,868 unique words, for each of which we have 17 years of count data, resulting in 11,268,756 word-year dyad observations.

The advantage of this research design is that our sample includes all trajectories, not just trajectories of concepts and phases that are known to once have been popular. We argue that such a sample have the distinct advantage of avoiding the selection bias common to the usual research design. The disadvantage of our research design is that we are unable to provide much contextual information about each word and trajectory studied. Because our sample includes many different words, we do not have detailed knowledge of how the concept underlying the words we study emerged and what kinds of actors that were associated with it. Thus, unlike studies of media mentions of quality circles, we cannot include controls for important events and actors (such as the activities of consultants, see [19]).

Another disadvantage of our sample is that it includes many words that some would argue should not be included in a risk set of concepts or words that could become popular. For example, our sample includes misspellings. An alternative approach would be to construct a much shorter list of concepts and words that represented cultural and management practices that were once proposed but perhaps did not take off. We agree that such an approach has its attractions but we also believe that constructing such a list is problematic and likely to lead to a selection bias, as it is very difficult to identify retrospectively the concepts that should or should not be in the risk set. A strength of our approach is that it does not require us to decide, in advance, whether a word or concept could become popular. For example, who is to say that what we now judge to be a misspelling might not in fact have become the dominant spelling?

Examining the most popular words in our sample (the words with the largest number of yearly mentions) illustrates that the way we constructed our sample does enable us to capture emerging phenomena. Three of the most popular words that emerged during the study period were “Qaeda”, “Taliban”, and “Lewinsky”, which all represent important phenomena. Other popular words include “iPod”, “eBay”, and “SARS”.

**Fig 1:**
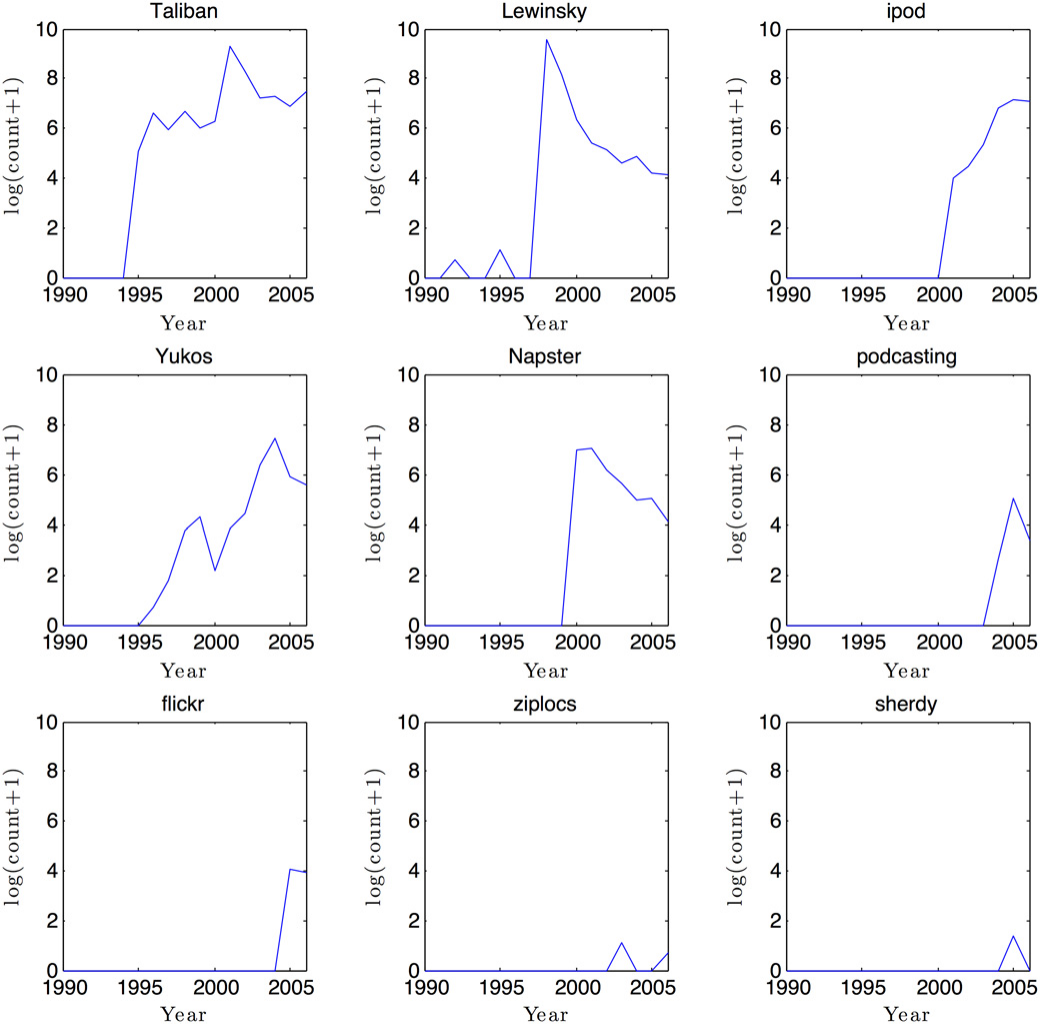
A few illustrative word trajectories from the New York Times archive.

**Fig 2:**
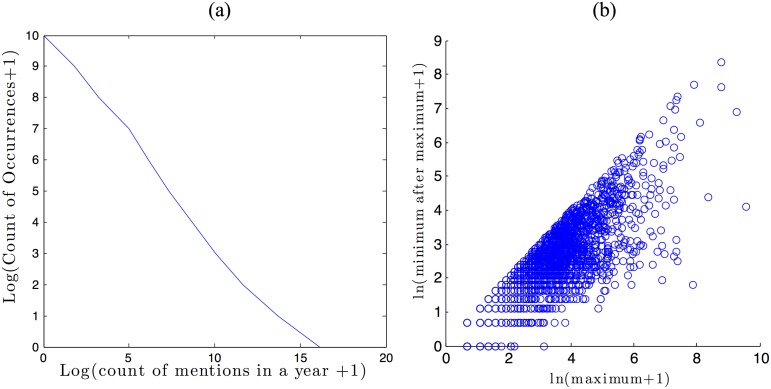
a: The distribution of word counts. b: Scatter plot with the maximums the trajectories reach and the minimum they reach after their peak.

Fig 1 shows nine words and their trajectories between 1990 and 2006. This figure is intended for illustration purposes and the words are not randomly selected. These graphs illustrate that word trajectories follow various patterns: increase and remain popular like “iPod” or “Taliban”; increase but then decrease, such as “Lewinsky” or “Napster”. Or, with the words “ziplocs” or “sherdy,” some words never really take off and remain around 0 or one mention and this is actually the most common pattern, as we illustrate in Fig 2b.

### Features of the Data

#### Skewed distribution

Fig 2a illustrates the distribution of the counts per year of the words in the sample on a log-log scale. The figure shows that this sample’s distribution plot follows a negative sloped line in a log-log scale, thus satisfies Zipf’s law [22]. The distribution of word counts is thus very skewed: most words are not mentioned at all or only mentioned a few times in a year, and only a handful of words in our sample are mentioned more than a few hundred times a year. In fact, 63.21% of the words in the New York Times data set receive only one mention, 8.12% five or more mentions, and only 3.28% of the words receive ten or more mentions a year. Thus, by studying phenomena that have become popular, one selectively samples an extreme tail of the distribution.

Fig 2b shows a scatter plot of the distribution of the maximum count reached by the words versus the minimum count reached after reaching the maximum count. These two statistics, maximum and minimum after reaching the maximum, are important because they help to identify the pattern of the trajectory of the words: (1) words that have a low maximum have never become popular; (2) words that have high maximum and high minimum after the maximum are the words that became popular and stayed so during the sample (such is “ipod” in Fig 1); and (3) words that have high maximum but low minimum after the maximum identify the fashion-like phenomena, that became popular but then went out of fashion, such as “Lewinsky”.

#### Word trajectories tend to decrease after reaching their maximum

We calculated the proportion of the maximum of the trajectory to the minimum reached after reaching the maximum. For most trajectories, we find a significant decrease: for 67% of the words that reach a maximum of ten or more mentions a year, the mentions later decrease 0; and for 87.21% of all words, trajectories decrease to a level that is less than 10% of the trajectory’s peak. Of course, the trajectory decreases by definition after reaching its maximum, but not necessarily by such a magnitude. The count of word mentions could theoretically stay high. Simulations of random trajectories (Poisson processes with a constant rate equal to 1.35; this rate implies that about 3.3% of the trajectories reach a level of 10 or more, similar to the proportion in the data) show that only 47% of the trajectories that reach at least 10 decrease to less than 10% of the maximum and only 19% of all trajectories decrease to a level that is less than 10% of their trajectory’s peak within 17 periods (the simulation is calibrated to 17 periods to reflect our data structure).

#### Word mentions are clustered in time

Finally, word mentions are clustered in time, indicating that word occurrences are not independent from each other. To test whether word clustering is indeed higher than would be expected from a random null-distribution, we rely on the scan test commonly used in the epidemiology literature [23–24]. The scan test compares the maximum of the sum of occurrences within a given time window (the time window is specified by the researcher, depending on the structure and size of the data) with what would be expected from uniformly distributed occurrences. The appendix explains how the scan test works.

The scan test does not have enough power to establish whether trajectories with very low counts are clustered. Thus, we focus on words that have at least ten mentions. From the sample of words that have at least ten mentions, 86.06% of the trajectories are significantly (at 5% level) more clustered than what would be expected from a uniform random baseline. From the subsample of words with at least a 100 mentions, 100% of the words are significantly clustered in time. These investigations confirm that most words are indeed clustered in time.

## The Effect of Selection Bias: Theory and Hypothesis

Our data show that most objects do not become popular and the popular ones may, or may not, become unpopular. Most research on fads and fashions, however, concentrates on a select set of practices and objects that once were popular such as quality circles. We argue that such a selection bias will lead to incomplete understanding of the drivers of popularity and of fads and fashions. To explore how selection bias can impact estimates of the drivers of popularity we here we review variables that have been proposed as predictors of popularity. Our discussion is not meant to provide a comprehensive review of the literature. Rather, our review focuses on variables that might have been under- or overestimated as a result of the tendency to focus on once popular objects. Because our purpose is to develop a quantitative model that can be estimated using our dataset, our review focuses on theories that incorporate variables we can measure using our data set. This implies that our review will ignore contextual factors and external events that impact popularity. While important, we cannot estimate their impact using our dataset.

### Past level of popularity

Many models of diffusion assume that the level of popularity in period t+1 is influenced by the level of popularity in period t. That is, a high level in period t predicts a high level in period t+1. To some extent, such a ‘level’ effect reflects inertia: people who have adopted a practice or acquired an object are unlikely to abandon it in the next period. If some proportion of the people who adopted the practice in the previous period do not reconsider their decision, researchers will observe a positive effect of past levels of popularity. Many scholars have argued that past level of popularity also has an effect on the probability that an agent who has not yet adopted the practice will do so. Such a positive ‘level’ effect on the adoption rate is implicit in most models of diffusion [25]. The idea is that the higher the number of current adopters, the higher the propensity to adopt a practice will be. The underlying mechanism is that non-adopters are more likely to be exposed to a popular object [26–28]. Popularity may also be an indication that the practice works well. Finally, people may find it more attractive to adopt a popular object [29–31], to coordinate their choices with others [8] because there is safety in numbers [32] or to avoid being seen as deviant [33–34].

Several well-known models of adoption and diffusion have examined the consequences of a positive level effect, including Coleman’s stochastic models of contagion [35], threshold models of collective behavior [32], and Barabasi and Albert’s model of preferential attachment [36]. These models assume that the probability of another adoption is an increasing function of the number of existing adopters and show that this assumption implies a skewed distribution of popularity where most practices reach a low level of popularity while a few become very popular [37].

Although most models of adoption have assumed a positive effect of past popularity, there are also theoretical arguments for a negative effect, at least for very popular practices. One reason is competition—the returns may decrease if many others have adopted a practice as emphasized by population ecologists [20]. Another reason is the desire to signal a unique identity [38]. Psychologists have proposed that although there is a desire to conform to norms, people also wish to distinguish themselves from others. In situations when competition or the desire to signal a unique identity is important, the effect of past popularity may thus be non-monotonic: initially positive but becoming negative for high levels of popularity [20].

How will the estimate of the effect of the level of past popularity be impacted by selectively sampling trajectories of objects that once were popular and then declined in popularity? [17] showed that if only popular practices were sampled, the effect of past popularity would be underestimated. We expect a similar underestimation if only trajectories of once popular objects that eventually became popular are sampled (we use the term “underestimation” when we expect the coefficient estimate to be lower in a select sample compared to the full sample).

### Changes in popularity

Potential adopters may be sensitive to changes in popularity in addition to the level of popularity [5, 12]. In particular, the propensity to adopt a practice may be an increasing function of the change in popularity. The idea is that practices that have recently risen in popularity are identified as the new and fashionable things to do and are more likely to be discussed and mentioned in media than practices that have a high level of popularity but have been around for some time. Individuals and organizations that want to be seen as fashionable are thus likely to adopt practices that have recently risen in popularity [2, 5]. Conversely, practices that have recently declined in popularity are viewed as going out of fashion and are dropped by fashion conscious past adopters and avoided by potential adopters.

It is not difficult to see how a positive effect of change in popularity can lead to rises and falls in popularity. A practice that increases in popularity will increase further in popularity. Increases in popularity thus lead to a process with positive feedback until there is a decrease in popularity that triggers further reductions in popularity.

The effect of past changes in popularity could also be negative: the propensity to adopt a practice might be a decreasing function of the change in popularity. Such an ‘inhibition’ effect could occur if potential adopters want to avoid practices and cultural objects that are currently fashionable. For example, [12] show that, controlling for the level of popularity, parents are less likely to pick names that have recently increased in popularity because such names are considered faddish. A desire to be a fashion-leader, rather than a follower, could also lead to a negative effect of changes in popularity: to avoid being seen as a follower, people may stay away from practices that have recently increased in popularity and instead bet on other practices that might increase in popularity in the future. A negative effect of changes in popularity will typically lead to oscillations: increases will tend to be quickly followed by decreases. To explain sustained periods of increases and decreases, a model in which people react to changes that have occurred over the several recent periods is needed.

How does selection bias impact estimates of the effect of the change in popularity? Using a select sample of trajectories of objects and ideas that once were popular but eventually became unpopular likely leads to overestimation of the effect of the change in popularity. The reason is that in these trajectories increases in popularity tend to be followed by increases and decreases by decreases.

### Age and Novelty Bonus

Popularity could also be a function of “age”, i.e., the time elapsed since the practice or object was first introduced or first adopted in the population. If there is a novelty bonus, popularity might decrease with the time since the first mention of an object. The underlying mechanism is that objects lose some of their attraction as time goes on, perhaps because individuals or organizations want to adopt novel practices and objects and shy away from older, “boring”, objects. A novelty bonus could explain the eventual decline of new practices. If the new practices happen to become fashionable, perhaps due to a positive level effect, they will eventually decline in popularity due to a novelty bonus.

How does selecting trajectories that all reach a relatively high level of popularity but then decline in popularity impact estimates of the effect of age? We suggest that the negative effect of age may be underestimated. The reason is that in a select sample of once popular concepts, most trajectories initially increase in popularity (because only trajectories that increase initially are likely to become popular and be included in the select sample). This rapid initial increase will coincide with low age, contributing to a spurious negative correlation between age and popularity.

### Competition and Limited Attention

Whether an object or practice becomes popular also depends on the popularity of other objects and practices. Objects may compete due to limited budgets or resources. Objects can also compete due to limited attention. Weng et al. [39] show that such competition for attention can explain the skewed distribution of popularity and persistence observed in online settings. Competition for attention could also possibly explain the increase and decrease in popularity. Suppose the popularity of a given object in the next period is a decreasing non-linear function of the maximum popularity achieved in the current period by competing objects. Specifically, suppose there is hardly any competition effect in the early periods when the popularity of the most popular object is low but there is a strong negative effect of the maximum popularity in later periods when the most popular object has reached a high level of popularity. Simulations show that a strong negative competition effect in the later periods can lead to a decline in popularity for objects that increased in popularity until then, thus generating faddish cycles of increases and decreases in popularity.

It is also possible that the popularity of other objects has a positive effect on the popularity of an object: if other objects become popular a given object may become more popular. The reason may be contagion in attention: a very popular book in an unusual genre may lead readers to discover other similar books. A positive effect of popularity of other objects may also simply indicate increasing attention to or demand for this class of objects.

What is the impact of selection bias on estimates of the effect of the popularity of other objects? In a selected sample, where most trajectories first increase and then decrease, the popularity of other objects will likely be overestimated: even if there is competition the popularity of other objects might even seem to have a positive effect on the popularity of a given object. The reason is that the popularity of a given object will be positively correlated with the popularity of other objects in a select sample because most trajectories follow the same pattern (increase and then decrease).

### Delays

Adopters may be sensitive to popularity not only in the most recent period but also in the more distant past [8, 20, 40–42]. Theoretically, such a delayed effect of past popularity is important because it can lead to cycles in popularity [8,42] that could explain why the popularity of some items waxes and wanes in a faddish manner. A delayed reaction to popularity may occur because of lags in responses: people may be stimulated to adopt a practice in period t, but may not be able to adopt it until several periods later [40–42]. Moreover, the level of popularity in the period in which an organization or individual first adopts a practice may have a permanent effect on the probability of continued adoption. For example, population ecologists have argued that the density of founding has a permanent effect on the exit rate of organizations (“density delay”, [20]). What is the effect of selectively sampling objects that once were popular and then declined in popularity on the estimate of popularity in the distant past? We expect that the effect of a selection bias is the same for popularity in the distant past as for past popularity: it will be underestimated in a select sample.

### Other Accounts

Several other important mechanisms have been proposed in the literature to account for popularity dynamics. One well-known explanation of faddish behavior assumes that the overall population can be divided into subgroups and different subgroups have different reactions to the level of popularity in their own and other subgroups. Specifically, individuals in low status groups are attracted to what is popular among high-status individuals but high-status individuals are deterred from what low-status groups find popular. This story, introduced by Simmel [11], has been discussed and proposed as an important mechanism for explaining faddish behavior by several well-known sociologists including Veblen [1], Coleman [35], and Bourdieu [9]. Formal models incorporating these ideas have also shown that this mechanism can produce cycles in popularity [43–45]. While this account is interesting, our data does not allow us to estimate the impact of popularity in different sub-groups. Other explanations of faddish behavior postulate a depletion effect, i.e., that the number of potential adopters decline over time. A depletion effect may occur due to ‘immunity’: an agent may become less likely to use an object this agent has once abandoned. Stochastic models in epidemiology show that a model with immunity can explain both a skewed distribution of popularity and why popularity rise and falls according to a faddish cycle [43–45]. It is difficult to estimate whether abandonment leads to immunity using our dataset, however, since we do not know who is using an object and hence cannot identify whether someone abandons an object.

## The Effect of Selection Bias: Simulations and Data

To examine the effect of selection bias more formally, we estimate a simple model of popularity dynamics using both simulation data and data from the New York Times archive. Using simulations we first explore how selection bias may impact coefficient estimates. We then use the data from the New York Times archive to estimate our model on the full data set as well as on a selected sample to empirically assess the impact of selection bias on coefficient estimates.

### 

#### Model Specification

To model the dynamics of popularity, we introduce a stochastic count model [5, 6]. Let the level of popularity in a period be Nt. In our data, popularity corresponds to the number of mentions in the New York Times during one year. Following [35], we assume that popularity can be modeled as a random variable drawn from a Poisson distribution. Formally,

$$P(N_{t}=n_{t})=e^{-\lambda (t)}\frac{\lambda {\left(t\right)}^{n}}{n_{t}!}$$ (1)

where *λ*(*t*) is the expected level of popularity in period t, E[N_t_].

We assume that the expected level of popularity depends on the past level of popularity, the change in popularity, age of an object, popularity of others, and popularity in more distant periods as follows:

$$\lambda \left(t\right)=\;exp\left[b_{1}+b_{2}\ln\left(N_{t-1}+0.1\right)+b_{3}\text{sign}\left(N_{t-1}-N_{t-2}\right)+b_{4}\ln\left(Prc_{t-1}\right)+b_{5}A_{t}+b_{6}\ln\left(N_{t-2}+0.1\right)\right]$$

Where sign(*N*_*t*-1_-*N*_*t*-2_) = 1 if *N*_*t*-1_>*N*_*t*-2_, sign(*N*_*t*-1_-*N*_*t*-2_) = 0 if *N*_*t*-1_ = *N*_*t*-2_, and sign(*N*_*t*-1_-*N*_*t*-2_) = -1 if *N*_*t*-1_<*N*_*t*-2_. Moreover, (*Prc*_*t*-1_) equals the popularity of the 10% most popular object in period *t-1* and *A*_*t*_ is the age of an object (the number of periods since it first obtained a popularity above zero).

Here *b*_1_ is a constant and *b*_2_ is the effect of (logged) past popularity. If *b*_2_ is positive, *N*_*t*-1_ has a positive effect on the expected level of popularity in period *t*. Because past popularity is often zero we add .1 to be able to take the logarithm. The parameter *b*_3_ determines the effect of past changes. If *b*_3_ is positive, there is a momentum effect: objects that have increased in popularity become more popular [5]. If *b*_3_ is negative, there is an inhibition effect: objects that have increased in popularity become less popular. We assume here that only the direction of past changes in popularity matters. The underlying idea is that people are sensitive to trends and increases, but the magnitude of the increase may be less relevant: an increase in popularity from 10 mentions to 15 may be as relevant as an increase from 100 to 150. In addition, working with the direction of the change makes the model less sensitive to outliers and ensures that large increases do not trigger run-away processes of increasing popularity. We get similar empirical results, however, if we estimate a model with the magnitude of the change.

The parameter *b*_4_ determines the effect of the popularity of others. Specifically, *b*_4_ determines the effect of *ln*(*Prc*_*t*-1_), where (*Prc*_*t*-1_) is the popularity of the 10% most popular object in the previous period. We use (*Prc*_*t*-1_) to capture the idea that the most popular objects get most attention. Other measures, using the sum of the popularity of all objects, give similar results. The parameter *b*_5_ determines the effect of the age of the object, *A*_*t*_, which is defined as the number periods since the object first obtained a popularity above zero. Finally, the parameter *b*_6_ determines the effect of the (logged) level of popularity two periods ago, *ln*(*N*_*t*-2_+0.1), which we use to capture a delayed effect (we again add 0.1 because *N*_*t*-2_ can be zero). Because our data consist of yearly counts of word mentions, we believe that longer delays are less realistic.

The proposed model is simplistic and does not take into account many of the variables and mechanisms that past literature has suggested. However, our purpose is not to develop the most comprehensive model of popularity dynamics but to examine theoretically and empirically how selection bias impact estimates of drivers of popularity. As a result, we focus on variables that we can measure using our data set.

#### Estimation Check

Suppose we had data that were generated by the model described above. One way to estimate the coefficients, and test whether the effect of past changes is significantly positive or negative, is to run Poisson regressions. Here we first use simulated data to examine whether one can recapture the underlying parameters of the model using Poisson regressions. A skeptic might doubt if this is possible since the past level of popularity is included both as a separate variable and in the term defining the sign of change. The model also includes popularity two periods back both as a separate variable and in the definition of the change variable. Finally, the dependent variable is endogenous since it depends on popularity in past periods. To examine any potential biases in estimating the coefficient, we ran Poisson regressions using simulated data from the above model. Specifically, we simulated 50000 trajectories each 15 periods long, and estimated a Poisson regression using the simulated data. Table 1 shows the parameter values we used in the simulations and the estimated coefficients. As shown, a Poisson regression is able to recover the underlying coefficients and provides unbiased estimates.

**Table 1:** Estimates of Poisson models using simulated data.

	True Coefficients	Estimates: All trajectories	Estimates: Max >27 & min after <4	Effect of Selection Bias
*b*_1_: Constant	1.00	.998	1.560	Overestimated
		(.0018)	(.018)	
*b*_2_: Level in period t-1	0.60	.598	.531	Underestimated
		(.0015)	(.012)	
*b*_3_: Change in Level	0.1	.101	.122	Overestimated
		(.0010)	(.006)	
*b*_4_: Popularity of Others	-0.05	-.049	-.047	Overestimated
		(.0003)	(.003)	
*b*_5_: Age	-0.1	-.099	-.130	Underestimated
		(.0003)	(.002)	
*b*_6_: Level in period t-2	0.3	.300	.272	Underestimated
		(.0013)	(.009)	
N		750000	7080	
Pseudo R2		.569	.623	
Log-likelihood		-1153324.4	-16875.587	

All parameter estimates are significant at the 1% level.

### Illustration: estimates on selected trajectories

To illustrate that the proposed model could offer a reasonable account of faddish behavior—if the typical data on practices that once were popular are used—we estimated the model on Abrahamson’s data on quality circles [2] and on David and Strang’s data on the spread of Total Quality Management [19]. [2] studied the popularity of quality circles by analyzing the number of articles in the management journals mentioning the expression “quality circle,” and found that quality circles followed a classical fashion pattern: the term has a very few mentions before 1979, it takes off in 1980, peaking in 1982 with 42 articles, then quickly declines to 25 articles in 1984 and only 13 articles in 1986. Similarly, [19] studied the count of articles in ABI/Inform that contained the expression “total quality management” or TQM, and also found an inverse U-shaped pattern.

Table 2 shows the estimated coefficients of the Poisson-regressions when we estimated our model on Abrahamson’s and David and Strang’s data. Because data on competing practices were not available, we did not estimate the effect of the popularity of other practices. The estimates show that there is a positive effect of (logged) past popularity, *b*_2_>0, as well as a positive effect of popularity two periods ago, *b*_6_>0. The effect of the change in popularity is positive, *b*_3_>0, suggesting a momentum effect. Finally, the effect of age is negative, *b*_5_<0, consistent with a novelty bonus.

**Table 2:** Estimates of Poisson models with previous level and sign of change, Abrahamson (1996 [2]) and David and Strang (2006 [19]) data.

	Abrahamson (1996)’s data	David and Strang (2006)’s data
*b*_1_: Constant	2.215*	2.650*
	(.315)	(.0261)
*b*_2_: Level in period t-1	.559*	.534*
	(.202)	(.137)
*b*_3_: Change in Level	.118	.182*
	(.139)	(.006)
*b*_5_: Age	-.189*	-.078*
	(.081)	(.017)
*b*_6_: Level in period t-2	.144	.074
	(.178)	(.117)
N	8	11
Pseudo R2	.480	.779
Log-likelihood	-25.655	-82.437

* Starred coefficient estimates are significantly different from zero at the 5% level.

**Fig 3:**
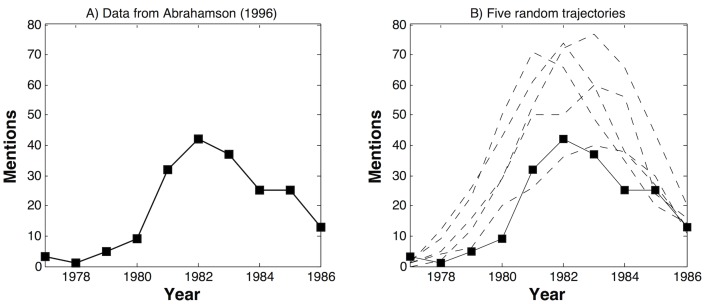
Abrahamson (1996)’s data: Comparison of the observed data and the predicted values from Poisson models using parameters from Table 2.

**Fig 4:**
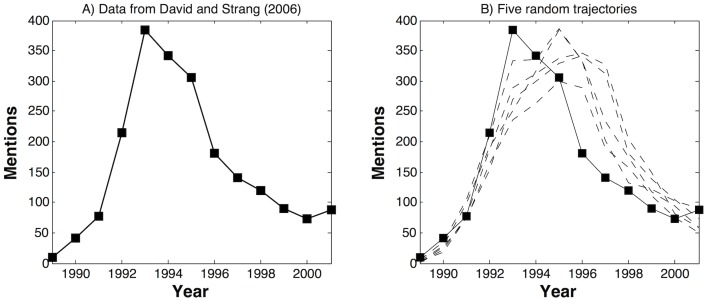
David and Strang (2006)’s data: Comparison of the observed data and the predicted values from Poisson models using parameters from Table 2.

The proposed model describes the data reasonably well: the Heckman pseudo-R2 values are 0.48 and 0.78 for the two data sets. To better show model fit, we plot the observed counts as well as five randomly generated trajectories based on the coefficient estimates. Fig 3 shows the result for Abrahamson’s data [2] on quality circles and Fig 4 shows the result for David and Strang’s data [19] on the spread of Total Quality Management. These randomly generated trajectories describe the observed counts well, although there is a tendency for the model to overshoot. Moreover, the model fails to predict the peak, which is not surprising: the exact location and timing of the peak is very hard to predict using diffusion models. As soon as the peak is reached, the model traces the data again.

While the estimated model reproduces the underlying pattern in the data, keep in mind that the data are selectively sampled: the researchers chose to study these practices because they were once popular. To illustrate that this selection bias can significantly distort coefficient estimates, we now turn to simulations.

## The effect of selection bias: Simulation results

What happens if researchers examine a selectively sampled set of trajectories of objects and practices that were once popular and subsequently declined in popularity instead of estimating models on a random sample of trajectories? To examine this, we estimated the above Poisson model using only a select sample of the 50 000 simulated trajectories. The subsample we selected consists of trajectories that reached a relatively high maximum but subsequently declined. Specifically, the subsample only included those trajectories that have reached more than 27 counts in a given year and, after reaching their maximum, declined to a level below 4 counts. This subsample constitutes about 1% of the full sample. The right-hand column of Table 1 shows the estimates for this selected subsample.

The coefficient estimates are clearly biased. The constant, *b*_1_, in strongly overestimated, the effect (logged) past popularity, *b*_2_, is underestimated, the effect of past changes, *b*_3_, is overestimated, the effect of the popularity of others, *b*_4_, is overestimated, the effect of age, *b*_5_, is underestimated, and the effect of popularity two periods ago, *b*_6_, is underestimated. Examination of the confidence intervals for the coefficient estimates in the selected sample shows that all the biases, except that for age, are significant: the 95% upper and lower confidence intervals for the coefficient estimates do not include the true parameter value. In contrast, the 95% upper and lower confidence interval for *b*_5_ does include the true value of the parameter, -0.05.

Overall, the results show that the common practice of selectively using a sample of trajectories that reached a high maximum before declining can provide a misleading impression of the dynamics of popularity. For example, the effect of past changes in popularity may be overestimated and the popularity of others might seem to have a more positive effect than what is actually the case. In fact, as other simulations show (not reported here), even if there is no effect of past changes in popularity, estimates using a selected sample may show a spurious positive effect. And even if there is no effect of the popularity of others, estimates using a selected sample may show a spurious positive effect.

## The effect of selection bias: Empirical results

The above results were based on simulations. Here we examine whether similar effects hold if we estimate the above Poisson regression models using our New York Times data. Our focus on words that did not appear before 1990 resulted in a sample of 662,868 unique words, and for each of these words we have 17 years of count data, yielding 11,268,756 word-year dyad observations. The left-hand column of Table 3 shows the result for the whole sample—in this regression all the observations are pooled, and standard errors are clustered for each word. In this regression we also include year, to control for possible trends in the number of words used in New York Times.

**Table 3:** Estimates of Poisson models using the New York Times Data.

	Estimates: All trajectories	Estimates: Max >54 & min after <7	Effect of Selection Bias
*b*_1_: Constant	-265.975***	-65.167***	Overestimated
	(4.603)	(23.596)	
*b*_2_: Level in period t-1	.714***	.339***	Underestimated
	(.015)	(.031)	
*b*_3_: Change in Level	-.238***	.111*	Overestimated
	(.0033)	(.060)	
*b*_4_: Popularity of Others	-.210***	-.058**	Overestimated
	(.012)	(.027)	
*b*_5_: Age	-.060***	-.071***	Underestimated
	(.004)	(.011)	
*b*_6_: Level in period t-2	.203***	.023	Underestimated
	(.018)	(.028)	
Year	.133***	0.034***	
	(0.002)	(0.012)	
N	11,268,754	16,609	
Pseudo R2	.364	.258	
Log-likelihood	-8645497.1	-328537.85	

***significant at the 1% level.

** significant at the 5% level.

* significant at the 10% level (robust standard errors clustered by trajectories).

The estimates from Poisson regression in Table 3 based on the full sample show that there is a positive effect of (logged) past popularity, *b*_2_>0, as well as a positive effect of popularity two periods ago, *b*_6_>0. The effect of the change in popularity is negative, *b*_3_<0, indicating an inhibition effect rather than a momentum effect. That is, increases in popularity do not lead to further increases but to a decrease in popularity. The effect of the popularity of others is negative, *b*_4_<0, consistent with a competitive effect. Finally, the effect of age is negative, *b*_5_<0, consistent with a novelty bonus.

The right-hand column of Table 3 shows the estimates for a selected sample of trajectories that initially increased in popularity but eventually decreased in popularity. The criteria for including a trajectory in this sample was that the maximum count was more than 54 (ln(count)>4) and that the count subsequently fell to a level below 7 (ln(count)<2). About 1% of the trajectories satisfy these criteria (977 words). The right-hand column of Table 3 shows the effect of this selection bias on the coefficient estimates. As shown, the constant, *b*_1_, is strongly overestimated. This effect is unsurprising, as we selected trajectories that reached a certain threshold. The effect of past popularity, *b*_2_, is underestimated, as predicted. Perhaps more interesting, the effect of past changes, *b*_3_, is strongly overestimated. Indeed, while the estimate of *b*_3_ is negative in the full sample, indicating an inhibition effect, the estimate of *b*_3_ is positive in the full sample (albeit not significant), suggesting a momentum effect. In other words, by only looking at trajectories that exhibit fashion like patterns (the selected sample), one might erroneously arrive at the conclusion that there is a momentum effect (*b*_3_>0), but this conclusion would be faulty and is merely due to a selection bias. The effect of the popularity of others, *b*_4_, is also overestimated. Thus, by looking at trajectories that exhibit fashion like patterns (the selected sample), one might erroneously arrive at the conclusion that there is not much of a competition effect. Following our simulation results, the effect of age, *b*_5_, is underestimated in the select sample. Finally, the effect of popularity two periods ago, *b*_6_, is underestimated. Examination of the confidence intervals for the coefficient estimates in the selected sample shows that all the biases, except that for age, are significant: the 95% upper and lower confidence intervals for the coefficient estimates do not include the coefficient estimates we found in the full sample. In contrast, the 95% upper and lower confidence interval for *b*_5_ does include the true value of the parameter. This is the same pattern we found in the simulation models above.

We also estimated Negative Binomial regressions, which allow for overdispersion. The signs of the coefficients and the pattern of the selection biases remain the same. Finally, we get similar results if we do not include words that were only mentioned once, thus eliminating words that have a count of zero during all periods except for in one period.

While the effect of selection bias is consistent with the simulation results, the biases are larger in the empirical data than in the simulations. For example, the estimate of *b*_3_—the effect of the change in popularity—increases substantially, and even reverses sign when going from the full sample to the selected sample in the empirical data. This large increase in the estimate of *b*_3_ suggests that the difference in the coefficient estimate between the full sample and the select sample is not only due to a statistical artifact, as in the simulation, but also to the fact that the effect of the change in popularity operates differently for objects that reached a high level of popularity. For example, it may be that the momentum effect is only important for objects that have reached a high level of popularity, because only such objects would be recognized as fashionable. Thus, the effect of the change in popularity may be contingent on the level of popularity reached. Similarly, the effect of the popularity of others may be contingent on the level of popularity reached: objects that are sufficiently popular may in fact benefit from the popularity of others.

## Discussion

Through simulations and the analysis of a data set that has not previously been used to analyze the rise and fall of cultural practices, we have shown that studying abandonment as well as including data on both popular and less popular practices, is essential for understanding the drivers of popularity. Specifically, we showed that by only focusing on practices that exhibit a fashion-like pattern, one might arrive at the erroneous conclusion about the effect of changes in popularity, the popularity of others, and the effect of age. In a select sample of faddish trajectories, the effect of change in popularity and the popularity of others are overestimated while the effect of age is underestimated.

Admittedly, the model we used in this paper is simplistic and does not take into account many of the variables and mechanisms which past literature has suggested. Still, our purpose was not to develop the most comprehensive model of popularity dynamics but to examine theoretically and empirically how selection bias impacts estimates of drivers of popularity. We encourage future researchers to extend our model to incorporate additional explanations for the dynamics of popularity, such as the possible dependencies among the diffusion of multiple products and practices [46, 47] or the network structure of the audiences adapting the innovations [48]. Similarly, future research should also investigate how adoption of child names, words or other fads might be different from the adoption of cultural practices [49].

Perhaps the most important extension of this research is to explore fad dynamics and possible selection biases using data on how many people or firms actually use a cultural or management practice. In this paper we have have used word mentions as a proxy for usage and interest. The use of media mentions was motivated by the fact that media mentions have been used in prior research and because it is possible to obtain a comprehensive dataset of media mentions that include less popular objects and practices. It may be possible, however, to track usage of all practices or objects in some domains, such as the adoption of online applications [40]. Using data from such a domain would also enable researchers to examine, in much more detail than we have done, the mechanisms driving popularity. Because we lack data on context and social networks, we could not examine, for example, whether the past level of popularity matters because people are more likely to be exposed to and hear about popular practices, through a word-of-mouth adoption process occurring within their local networks, or whether a positive effect of past popularity simply reflects inertia. As a result, our data and result cannot help to shed light on important debates within the diffusion literature about the relative importance of, for example, social learning versus contagion [10, 15, 29, 35]. Studies using more detailed data could address whether selection biases impacts estimates of the importance of alternative mechanisms.

Still, our findings have important implications for scholars and practitioners studying the dynamics of popularity. For scholars, the findings underscore the need to examine a more complete range of popularity that includes both popular and unpopular practices and pay more attention to constructing the risk set of possible trajectories in order to better understand the dynamics of popularity. Researchers who concentrate on faddish trajectories, to learn more about the determinants of fads, may miss the importance of competition and novelty. The findings also suggest that the idea of momentum—a positive effect of the change in popularity—may not be as important in explaining fads and fashions as previously thought. Researchers who concentrate on faddish trajectories may indeed find evidence for a positive effect of changes in popularity—a momentum effect. As we have shown, such a finding may be spurious and an artifact of a sample selection bias. Our analysis of the data suggests that the opposite mechanism operates: increases in popularity lead to reductions rather than increases in popularity.

What explains the observed negative effect of the change in popularity? One possibility is that people want to avoid objects that are evaluated as fashionable and increases in popularity is used as an indication of fashion. A simple alternative explanation for the observed the negative effect is that the probability of abandoning a practice is higher for novice adopters. That is, an individual who has engaged in activity A during periods 1,2,3, and 4 is less likely to abandon A in period 5 than an individual who started doing A in period 4. This assumption is sufficient to explain why the expected number of individuals or organizations that abandon the practice in the next period is larger when there is a large number of novice adopters present, which in turn is associated with a large increase in popularity. To explain this, suppose the popularity of an object increases from 70 in period 3 to 100 in period 4. Thus there are at least 30 novice adopters (individuals who did not engage in A in the previous period). Since novice adopters have a high rate of abandonment in period 5, popularity might fall even if there is a positive effect of the level of popularity on the propensity to adopt for those who do not currently engage in the activity. Compare this to a situation when the level of popularity is 100 in period 4 and it was 99 in period 3. Most likely, there were fewer novice adopters in period 4 in this scenario and the average rate of abandonment is thus lower.

If this interpretation of the effect of change in popularity is correct, the implications for how to make a novel concept or practice popular are very different. A positive effect of change in popularity suggests that fast increases in popularity lead to further increases in popularity, meaning that recruiting more adopters is likely to increase the chances of a novel concept becoming popular. If the effect of past changes is negative, and the reason is the mechanism we have proposed, a better strategy might be to ensure continued adoption by organizations and individuals that have already adopted the novel practice. Continued adoption leads to a lower abandonment rate. More generally, to ensure a high level of popularity, practitioners need to strike a balance between recruiting new adopters and spending resources to ensure the continued adoption of existing adopters.

## Appendix: Scan Tests

To illustrate how the scan test is calculated, consider the word “iPod” with the following count of mentions from 1990 to 2006, clearly clustered in time: 0, 0, 0, 0, 0, 0, 0, 0, 0, 54, 88, 206, 924, 1311, 1174 (see Fig 1 for the plot of this trajectory). For this illustration, we choose the time window to be three, and the maximum of three consecutive years’ count is 3,409, which is the sum of the counts in 2004–6. What would be the maximum of the sum of three consecutive years with the assumption that the words are uniformly distributed? To generate the null-distribution, Monte Carlo simulations are conducted. Imagine that that each of the 17 years is represented by a bin, and there are 3,757 balls (the mentions of “iPod”), and we place each ball in a bin randomly chosen. The maximum of the sum of the three consecutive bins represents the count of what would be expected under a uniform null. We do 10,000 simulations, and find that in all 10,000 simulations the observed maximum, 3,409, is higher than the simulated maximum, therefore we can reject the null-hypothesis of no-clustering at 0.01%. In other words, the trajectory of the word “ipod” is more clustered than what would be expected from a random baseline.

To provide an overall sense of clustering on the whole sample of 662,868 words, we calculate the scan statistics individually (which involves calculating the null distribution for each word and comparing it to the observed value). Before discussing the results, we note that for most words with a very low count it is impossible to get a significant result. For example, with a trajectory 0,0,0,0,1,0,0 the observed maximum of one always equals the simulated value. In other words, the scan test does not have enough power to establish whether trajectories with low counts are clustered. To deal with this problem, we focus on words that have at least ten mentions and we have reason to believe that these words would not be representative for the whole sample.

For the sample of words that have at least ten mentions, 86.06% of the trajectories are significantly (at 5% level) more clustered than what would be expected from a uniform random baseline. For the remainder (13.94%), the test cannot assess significance. Then, to further increase the power of the test, we focused on the subsample of words with at least a 100 mentions and found that all 100% of the words are significantly clustered in time. These investigations confirm that most of the words in the sample are indeed clustered in time.
